# Neuroblasts migration under control of reactive astrocyte-derived BDNF: a promising therapy in late neurogenesis after traumatic brain injury

**DOI:** 10.1186/s13287-022-03232-0

**Published:** 2023-01-05

**Authors:** Na Wu, Xiaochuan Sun, Chao Zhou, Jin Yan, Chongjie Cheng

**Affiliations:** 1 Department of Neurosurgery, The First Affiliated Hospital of Chongqing Medical University, No.1 of Youyi Road, Yuzhong District, Chongqing, 400010 China; 2 Department of Pediatric Surgery, Chongqing University Three Gorges Hospital, Wanzhou District, Chongqing, China

**Keywords:** Traumatic brain injury, Neuroblast, Neuronal migration, Brain-derived neurotrophic factor, CC chemokine ligand 2, Monocyte chemoattractant protein-1

## Abstract

**Background:**

Traumatic brain injury (TBI) is a disease with high mortality and morbidity, which leads to severe neurological dysfunction. Neurogenesis has provided therapeutic options for treating TBI. Brain derived neurotrophic factor (BDNF) plays a key role in neuroblasts migration. We aimed to investigate to the key regulating principle of BDNF in endogenous neuroblasts migration in a mouse TBI model.

**Methods:**

In this study, controlled cortical impact (CCI) mice (C57BL/6J) model was established to mimic TBI. The sham mice served as control. Immunofluorescence staining and enzyme-linked immunosorbent assay were performed on the CCI groups (day 1, 3, 7, 14 and 21 after CCI) and the sham group. All the data were analyzed with Student’s *t*-test or one-way or two-way analysis of variance followed by Tukey’s post hoc test.

**Results:**

Our results revealed that neuroblasts migration initiated as early as day 1, peaking at day 7, and persisted till day 21. The spatiotemporal profile of BDNF expression was similar to that of neuroblasts migration, and BDNF level following CCI was consistently higher in injured cortex than in subventricular zone (SVZ). Reactive astrocytes account for the major resource of BDNF along the migrating path, localized with neuroblasts in proximity. Moreover, injection of exogenous CC chemokine ligand 2 (CCL2), also known as monocyte chemoattractant protein-1, at random sites promoted neuroblasts migration and astrocytic BDNF expression in both normal and CCI mice (day 28). These provoked neuroblasts can also differentiate into mature neurons. CC chemokine ligand receptor 2 antagonist can restrain the neuroblasts migration after TBI.

**Conclusions:**

Neuroblasts migrated along the activated astrocytic tunnel, directed by BDNF gradient between SVZ and injured cortex after TBI. CCL2 might be a key regulator in the above endogenous neuroblasts migration. Moreover, delayed CCL2 administration may provide a promising therapeutic strategy for late neurogenesis post-trauma.

**Supplementary Information:**

The online version contains supplementary material available at 10.1186/s13287-022-03232-0.

## Highlights

1. Neuroblasts migrated along the activated astrocytic tunnel, directed by BDNF gradient between SVZ and injured cortex after TBI. CCL2 might be a key regulator in the above endogenous neuroblasts migration.
2. Exogenous CCL2 promoted neuroblasts migration and reactive astrocytes-derived BDNF expression not only in normal mice but also in mice on day 28 after CCI.
3. Notably, these neuroblasts also possessed the potential to differentiate into mature neurons.

**Supplementary Information:**

The online version contains supplementary material available at 10.1186/s13287-022-03232-0.

## Background

Traumatic brain injury (TBI) is a disease with high mortality and morbidity, which leads to severe neurological dysfunction, such as severe motor, neural psychological, and cognitive disabilities [1–4]. Despite extensive researches, there is still no ideal treatment for TBI due to multiple complications during pathogenesis [2, 4].

Lost neurons in adult brain could not be replaced was once a neurological dogma in the nineteenth century. However, this belief was subverted when postnatal and adult stem cells from the mammalian subventricular zone (SVZ) and subgranular zone (SGZ) had been rediscovered [5]. Up to now, stem cell therapy and neurogenesis have provided therapeutic options for treating TBI [4, 6].

In the adult brain, immature neurons called neuroblasts are continuously generated in the SVZ [7–9]. These neuroblasts migrate rapidly through the rostral migratory stream (RMS) to the olfactory bulb, where they mature and are integrated into the neuronal circuit [8]. After brain injury, some of the neuroblasts in the SVZ migrate toward the site of injury to repopulate the injured tissues [5, 10, 11]. Lee et al. [12] described that the neural progenitors in ischemic striatum were significantly increased on days 5 and 7 post-subarachnoid hemorrhage. Grade et al. [13] reported that many neural progenitors migrated from the SVZ into ischemic area at 2 weeks after ischemic stroke. In aspiration lesion model, neuroblast migration started 2 days post-lesion, and this migration appeared to be persistent even 2 months after lesion [10]. Apparent discrepancy in these previous studies might arise from differences in lesion models. So far, the spatiotemporal profile of neuroblasts migration following TBI in a controlled cortical impact (CCI) model remains largely unknown.

The notable migratory capacity of SVZ-derived neuroblasts is essential for efficient neuronal regeneration in remote areas of the brain. As these neurons migrate for long distances through adult brain tissues, they are supported by various guidance cues (Brain derived neurotrophic factor (BDNF) [5, 9, 12–19], Vascular endothelial growth factor (VEGF) [9, 19], Angiopoietin-1(Ang-1) [9, 19], Stromal cell derived factor-1 alpha (SDF-1*α*) [5, 9, 19–21]) as chemoattractants. BDNF and its receptors are found to play important roles in the guidance of neural migration [14, 16, 19, 22, 23]. However, it is unclear how BDNF affects the migration of neuroblasts after TBI.

In the present study, we established CCI mice models to investigate (i): the pattern and mechanism of endogenous neuroblasts migration after TBI; (ii): the key regulating principle of BDNF in endogenous neuroblasts migration after TBI; (iii): the reprogramming of neuroblasts migration by artificial intervention on such steps was further attempted for clinical treatment of TBI, especially late phase TBI.

## Material and methods

### Animals

A total of 483 adult male C57BL/6J mice, aged 8–12 weeks and weighing 20–25 g, were purchased from the Experimental Animal Center of Chongqing Medical University [Chongqing, China; license No. SYXK-(Yu)-2018-0003]. All mice were housed in a standard animal facility under controlled temperature (21 °C) and photoperiod (12 h light/12 h dark) with food and water available ad libitum. All experiments were approved by the Chongqing Medical University Administrative Panel on Laboratory and the Ethics Committee of the First Affiliated Hospital of Chongqing Medical University. All experiments were designed and reported in accordance with the Animal Research: Reporting of In Vivo Experiments (ARRIVE) guidelines. All surgeries were performed under anesthesia, and mice were anesthetized by inhalation with 3% isoflurane (flow rate 3 L/minute, Yuyan Instruments, Shanghai, China) in 67% N_2_O/30% O_2_ until they did not respond to a tail pinch. Then, 1.5% isoflurane (flow rate 0.5 L/min) was used for anesthesia maintenance. Throughout the experiment, the feeding and killing of animals met the requirements of scientific research ethics, maximize the safety and avoid unnecessary harm of the animals.

### Experimental design

The experimental process is shown as follows. Randomization was performed in the grouping of mice, according to the table of random numbers. The investigators were blinded to the experimental approaches and further analysis.

#### Experiment I

To investigate the spatiotemporal profile of neuroblasts migration after TBI, the mice were used to establish controlled cortical impact (CCI) models of TBI as previously described [3, 24]. Immunofluorescence staining of anti-doublecortin antibody (DCX, a marker of neuroblasts) was performed on the CCI groups (day 1, 3, 7, 14 and 21 after CCI) and the sham group (*n* = 6/per group).

#### Experiment II

To explore the role of BDNF in the orientation of neuroblasts migration, immunofluorescence staining of anti-BDNF antibody and enzyme-linked immunosorbent assay were performed on the CCI groups (day 1, 3, 7, 14 and 21 after CCI) and the sham group (*n* = 6/per group).Then, brain sections from CCI mice were immunostained with anti-BDNF, anti-NeuN (a marker of mature neuron), anti-GFAP(a marker of reactive astrocyte), anti-Iba-1 (a marker of microglia), and anti-CD31 (a marker of endothelial cell) antibodies to identify which antibody dominates the expression of BDNF within migrating path.

#### Experiment III

We attempted to seek potential regulators of neuroblasts migration with following criteria: polyergic control of astrocyte activation and BDNF secretion, by reviewing related literatures [25–40]. We found four cytokines [CC chemokine ligand 2 (CCL2), also known as monocyte chemoattractant protein-1(MCP-1), TNF-*α*, IL-6 or IL-1*β*]. Then, a series of experiments were carried out. Firstly, exogenous CCL2, TNF-*α*, IL-6 or IL-1*β* at different concentrations (50 ng/mL, 100 ng/mL and 500 ng/mL; 2 μl/per mouse) were injected with 5-µl needle into the cortex (coordinates: anterior–posterior 4 mm; medial–lateral 3 mm; dorsal–ventral 2–3 mm) of normal mice. Immunofluorescence staining of anti-DCX, anti-GFAP, anti-NeuN and anti-BDNF were performed on day 3, 7 and 14 after injection (*n* = 6/time point). The exogenous chemoattractant CCL2 showed maximal effects to provoke neuroblast migration. Next, CCL2 with the optimum concentration was injected into the cortex near lesion in mice on 28 days after CCI. Immunofluorescence staining of anti-DCX antibody, anti-GFAP and anti-BDNF were performed on day 7 after injection (*n* = 6/time point).

#### Experiment IV

To identify the spatiotemporal character of CCL2 after TBI, immunofluorescence staining of anti-CCL2 antibody and enzyme-linked immunosorbent assay were performed on the CCI groups (day 1, 3, 7, 14 and 21 after CCI) and the sham group (*n* = 6/per group).

#### Experiment V

To test whether blocking CCL2/CCR2 signaling pathway can attenuate the above neuroblasts migration after TBI, mice were divided into the TBI group and TBI-CCR2 antagonist group (*n* = 6/per group). Immunofluorescence staining of anti-DCX antibody was performed on day 7 after CCI.

### Drugs

PF-4136309, a CC chemokine ligand receptor 2 (CCR2) antagonists (0.05 mg/mL; MCE, Shanghai, China), was first intraperitoneally injected pre day 1 before CCI, at a dose of 0.3 mg/kg, and was then injected daily for 5 consecutive days after CCI.

### Controlled cortical impact (CCI)

As previously described [3, 41, 42], CCI was performed to mimic moderate TBI in the right parietal cortex and underlying hippocampus, with pronounced behavioral deficits but virtually no mortality. A circular craniotomy (5 mm in diameter) was performed at 2.0 mm posterior to bregma and 1.0 mm lateral from the sagittal suture over the right parietal cortex. Following the craniotomy, a CCI model was established with a TBI-0310 TBI model system (Precision Systems and Instrumentation, USA) and the impact parameters were set as follows: 5.0-m/second velocity, 100-ms dwelling time, 2.0-mm depth and 3.0-mm diameter impactor. The sham mice underwent craniotomy without impact. Mice maintained normal body temperature throughout the entire procedure. The rate of mice survival was 99% after CCI.

### Immunofluorescence staining

Immunofluorescence was performed according to previous studies [2]. In brief, mice were killed on days 1,3,7,14, and 21 after CCI, and perfused with PBS and 4% paraformaldehyde. The collected brains were postfixed in 4% paraformaldehyde overnight at 4 °C, and then were cryoprotected in graded sucroses (10%, 20%, and 30%). Next, the brains were embedded in optimal cutting temperature compound and cut into 15 μm frozen coronal sections. Slides were washed, treated with antigen retrieval, and blocked with 5% bovine serum albumin at room temperature for 60 min. The sections were then incubated overnight at 4 °C with primary antibodies, including rabbit polyclonal anti-doublecortin (DCX; 1:100; Abcam; a marker of newly generated immature neurons called neuroblasts), goat monoclonal anti-doublecortin (DCX; 1:100; Novus Biologicals), mouse monoclonal anti-doublecortin (DCX; 1:100; Santa Cruz), mouse monoclonal anti-GFAP (1:400; BD Biosciences; a marker of reactive astrocytes), mouse monoclonal anti-ionized calcium-binding adaptor molecule 1 (Iba1; 1:25; GeneTex; a marker of microglia), mouse monoclonal anti-neuronal nuclear antigen (NeuN; 1:200; Novus Biologicals, Littleton, NH, USA; a marker of mature neurons), mouse monoclonal anti-CD31 (1:100; Abcam; a marker of endothelial cells), rabbit monoclonal anti-BDNF (BDNF; 1:100; Abcam), and rabbit CC chemokine ligand 2 (CCL2; 1:100; Abcam). Slides were then washed and incubated at room temperature for 60 min with the appropriate secondary antibodies: IFKine™ Red Donkey Anti-Goat IgG (1:200; Abbkine), goat anti-rabbit DyLight 594 (1:200; Abbkine), goat anti-mouse DyLight 488 (1:200; Abbkine), goat anti-rabbit DyLight 488 (1:200; Abbkine), goat anti-mouse DyLight 594 (1:200; Abbkine), and goat anti-mouse DyLight 405 (1:200; Beyotime Biotechnology). Cell nuclei were stained with 4′,6-diamidino-2-phenylindole (DAPI; Sigma-Aldrich). Images were captured using a fluorescence microscope (Leica, Wetzlar, Hesse, Germany).

### Microscopical analysis and image quantification

Lesion cortex was screened for 15-µm coronal sections at − 0.9,  − 0.6, + 0.0, + 0.6, + 0.9 and + 1.2 mm from bregma. To maintain consistency for image analysis, the same imaging threshold and exposure time were applied for each condition and each time point. Approximately 4–5 randomized images at 20 × magnification in the region of interest (ROI) (ROI: DCX-positive cells in CC and cortex; BDNF-positive cells in peri-lesion cortex or in peri-SVZ; BDNF/GFAP-positive cells or CCL2-positive cells in the cortex) were captured from each coronal section. The immune-positive cell numbers were calculated with ImageJ software (NIH, Bethesda, MD, USA) and were presented as the mean number of cells per square millimeter. The results were further analyzed using GraphPad Prism 8.0.1.

### Enzyme-linked immunosorbent assay

Tissue samples were weighed immediately after collection to obtain the wet weight, snap frozen in liquid nitrogen and stored at − 80 °C until analysis. Each sample was transferred to ice-cold homogenization buffer and homogenized for 1 min in a tissue homogenizer. The lysate from each sample was centrifuged at 5000 g for 20 min at 4 °C and the supernatant solutions were collected. The supernatant from each sample was frozen for subsequent measurements of ELISA kit (MEIKE, Jiangsu, China) following the manufacturer’s protocol. In brief, 50 µL of standards or 50 µL samples were added into each flat-bottom wells, covered with an adhesive strip, and incubated for 120 min at 37 °C. Wells were washed 3 times with 300 µL of diluted wash buffer. Diluted biotinylated mouse anti-BDNF or anti-CCL2 monoclonal antibody (100 µL) was added to each well, covered with an adhesive strip, and incubated for 60 min at 37 °C. Wells were washed again 3 times with 300 µL of diluted wash buffer. Dilution of streptavidin-HRP conjugate solution (50 µL) was added to each well, covered with an adhesive strip, and incubated for 20 min at room temperature. Wells were washed 3 times with 300 µL of diluted wash buffer. About 50 µL of substrate solution was added to each well and incubated at 37 °C for 15 min. Reaction was stopped by adding 50 µL of stop solution to each well. The wells were read immediately using ELISA plate reader at 450 nm. Optical density (OD) of standard solution was plotted against known concentration of the standards to get the standard curve. Unknown concentration of BDNF or CCL2 in the samples was calculated by plotting their OD values into the standard curve. Finally, data were expressed as pg/g tissue and group mean was determined.

### Statistical analysis

PASS software was used to predetermine the sample sizes. No animals or data points were excluded from the analysis. All the data are presented as mean ± standard error of mean (SEM). All statistical analyses were performed with GraphPad Prism 8.0.1 software (GraphPad Software, San Diego, CA, USA). All the data were analyzed with Student’s *t*-test or one-way or two-way analysis of variance followed by Tukey’s post hoc test. *P* values < 0.05 was considered statistically significant.

## Results

### Spatiotemporal profile of neuroblasts migration after TBI

**Fig. 1:**
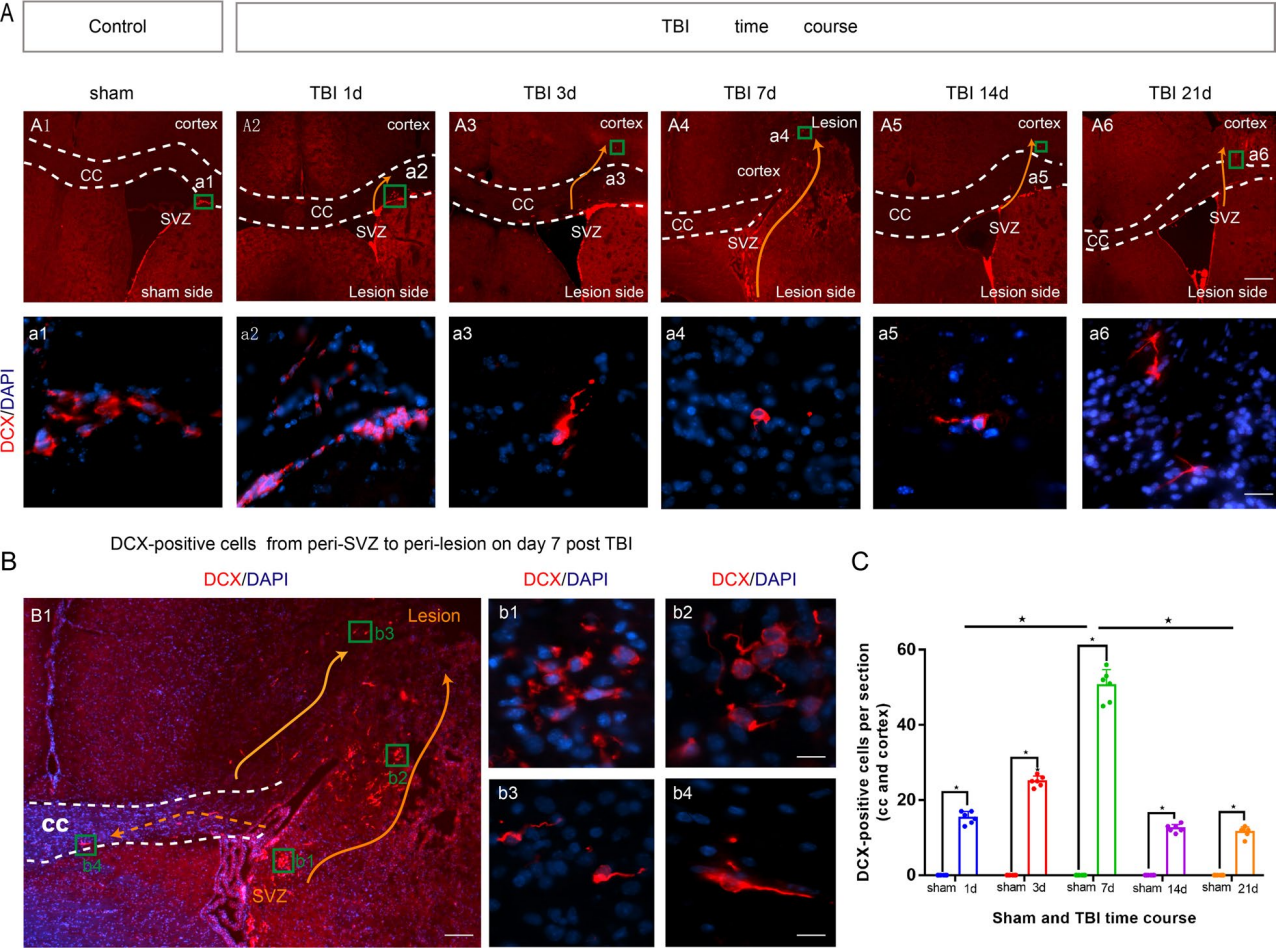
Spatiotemporal characteristics of neuroblasts migration after TBI. Coronal sections of forebrain were immunostained with anti-DCX antibody (red, neuroblasts) and DAPI (blue, nucleus). The level of neuroblast migration from SVZ into the CC and the perilesional cortex was determined by DCX/DAPI co-staining. **A** photomicrograph showing the distribution of DCX^+^ cells in the CCI groups (1, 3, 7, 14 and 21 days after CCI) and the sham group. The DCX-positive cells were found in CC on day 1 post-CCI, and in cortex on day 3, day 14 and day 21 post-CCI. The DCX-positive cells were present in lesion cortex on post-CCI day 7. In the sham group, DCX positive cells were only observed in SVZ. Scale bar = 5 μm (**A1**–**A6**), Scale bar = 125 μm (**a1**–**a6**). **B** Images of DCX^+^/DAPI^+^ cells on day 7 post-CCI. DCX positive cells were observed as spherical clusters (**b1**) or assembled in chains (**b2**) or individual cells (**b3** and **b4**). Scale bar = 5 μm (**B1**), Scale bar = 125 μm (**b1**–**b4**). **C** The number of migrating cells (DCX^+^ of DAPI^+^ cells) in the CC and cortex at different CCI time courses (1, 3, 7, 14 and 21 days after CCI) and sham control. The number of DCX-positive cells increased above the baseline (sham control) level on day 1 and 3 post-CCI and increased significantly above the baseline on day 7 post-CCI (*P* < 0. 05). The number of DCX-positive cells declined on day 14 and 21 post-CCI, but still above the baseline (sham control) level (*P* < 0.05). Data are expressed as the mean ± SEM, *n* = 6 for each time-point. *, *P* < 0.05; one way ANOVA with Tukey’s multiple comparisons test. CCI: controlled cortical impact; TBI: traumatic brain injury; SVZ: subventricular zone; DAPI: 4,6-Diamidino-2-phenylindole; CC: corpus callosum

Compared with the sham group, DCX-positive cells were found in SVZ and corpus callosum (CC) on day 1 post-TBI, in cortex on day 3 post-CCI, in lesion area on day 7 post-CCI, indicating the orient migration of neuroblasts from SVZ to lesion(Fig. 1A, B). The number of DCX positive cells in CC and injured cortex peaked on day 7 post-CCI (*P* < 0. 05) (Fig. 1A–C), and then decreased on day 14 and 21 post-CCI (Fig. 1C).

### BDNF gradient is responsible for the navigation of neuroblasts after TBI

**Fig. 2:**
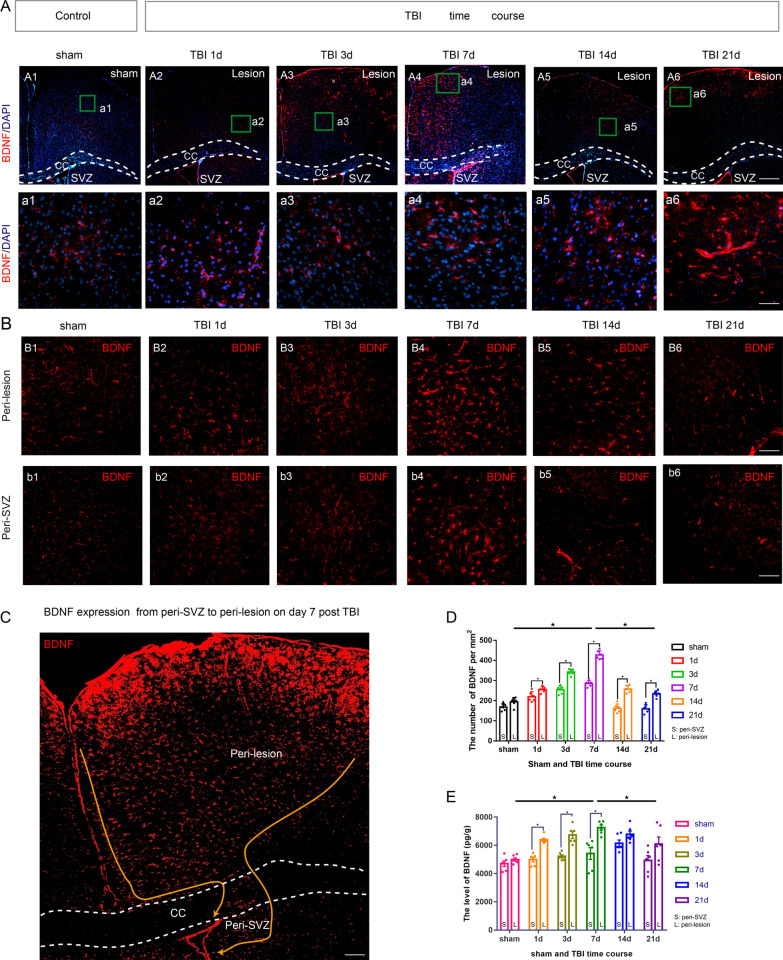
Spatiotemporal characteristics of BDNF expression after TBI. Coronal sections of forebrain were immunostained with anti-BDNF antibody (red, BDNF-positive cells) and DAPI (blue, nucleus). The levels of BDNF in peri-SVZ and in peri-lesion cortex were determined by BDNF/DAPI co-staining and ELISA analysis. **A** photomicrograph showing the distribution of BDNF+ cells at different CCI time courses (1, 3, 7, 14 and 21 days after CCI) and sham control. We observed the BDNF-positive cells in the cortex nearby CC increased on day 1 post-CCI, and significantly increased on day 3. There was a peak expression on day 7 post-CCI. The BDNF-positive cells showed a downward trend on day 14 and on day 21 post-CCI. Scale bar = 5 μm (**A1**–**A6**), Scale bar = 50 μm (**a1**–**a6**). **B** photomicrograph showing the distribution of BDNF+ cells in peri-SVZ and peri-lesion at different CCI time courses (1, 3, 7, 14 and 21 days after TBI) and sham control. The number of BDNF-positive cells was significantly larger in peri-lesion area than in peri-SVZ area at different time courses post-CCI. Scale bar = 25 μm. **C** Images of BDNF+/DAPI+ cells on day 7 post-CCI. **D** The number of migrating cells (BDNF+/DAPI+ cells) in peri-SVZ and peri-lesion cortex at different CCI time courses (1, 3, 7, 14 and 21 days after CCI) and sham control. The number of BDNF-positive cells increased markedly relative to the baseline (sham control) in peri-lesion area on 7 days post-CCI. The number of BDNF-positive was markedly higher on 7 days than on 21 days post-CCI. **E** ELISA analysis was applied to measure the concentration of BDNF in peri-SVZ and peri-lesion cortex at different CCI time courses (1, 3, 7, 14 and 21 days after CCI) and sham control. The level of BDNF expression was significantly higher in peri-lesion area than in peri-SVZ area at different time courses post-CCI. Data are expressed as the mean ± SEM, *n* = 6 for each time-point. *, *P* < 0.05; two-way ANOVA with Tukey’s multiple comparisons test. CCI: controlled cortical impact; TBI: traumatic brain injury; SVZ: subventricular zone; DAPI: 4,6-Diamidino-2-phenylindole; CC: corpus callosum

BDNF-positive cells in the migrating path of neuroblasts increased on day 1 and 3 post-CCI (Fig. 2A, B), reaching a peak expression on day 7 (Fig. 2A–C), and showed a downward trend on day 14 and 21 (Fig. 2A, B). The number of BDNF-positive cells and the level of BDNF expression were significantly higher in peri-lesion area than in peri-SVZ area at different post-CCI time points (*P* < 0. 05) (Fig. 2D, E).

### Reactive astrocyte dominates the expression of BDNF within migrating path after TBI

NeuN, GFAP, Iba-1 and CD31 were colocalized with BDNF respectively (Additional file 1: Fig. S1 A–D). Reactive astrocytes were found to be a major factor stimulating BDNF expression after CCI (*P* < 0. 05) (Additional file 1: Fig. S1 A, E).

**Fig. 3:**
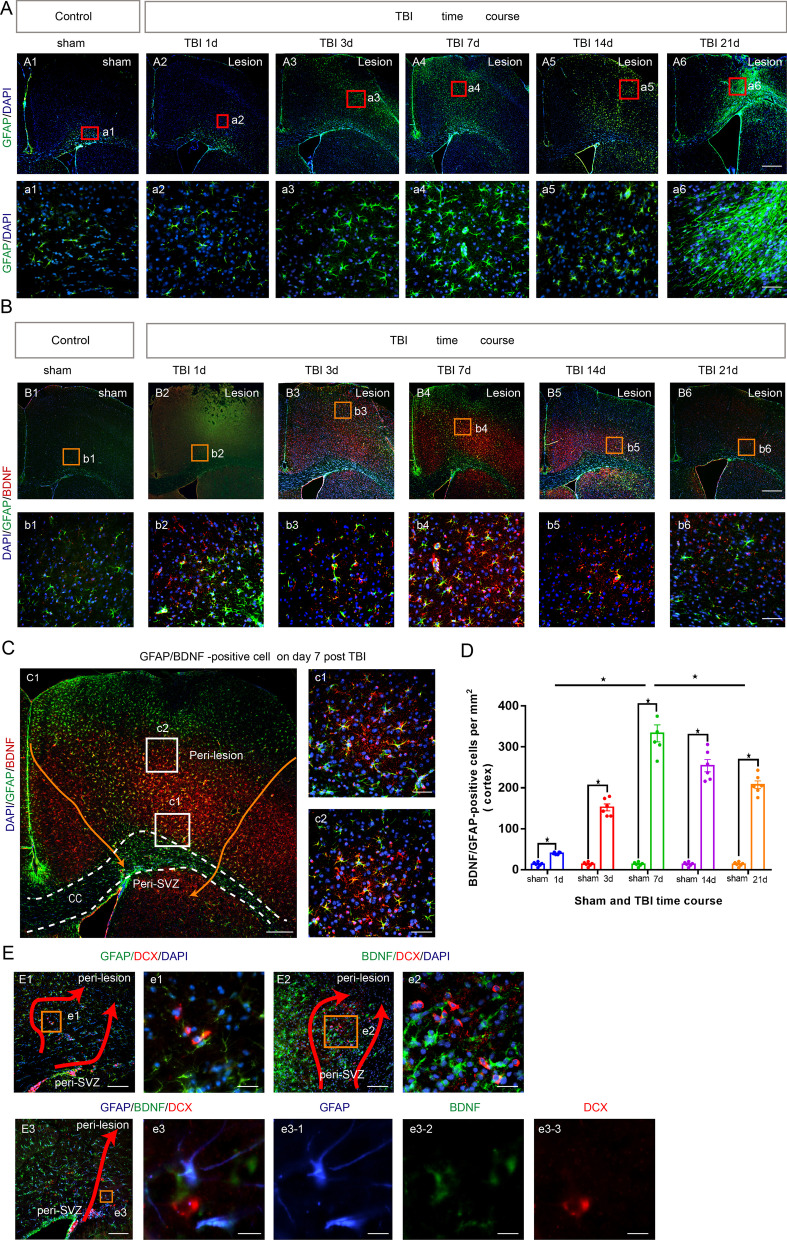
Spatiotemporal characteristics of reactive astrocytes and reactive astrocyte-derived BDNF after TBI, and close relationship with migrating neuroblasts. Coronal sections of forebrain were immunostained with anti-GFAP antibody (green, reactive astrocytes), anti-BDNF antibody (red or green, BDNF-positive cells) and DAPI (blue, nucleus). **A** photomicrograph showing the distribution of GFAP ^+^ cells at different TBI time courses (1, 3, 7, 14 and 21 days after TBI) and sham control. Scale bar = 5 μm (**A1**–**A6**), Scale bar = 50 μm (**a1**–**a6**). **B** photomicrograph showing the distribution of BDNF^+^/GFAP ^+^ cells at different CCI time courses (1, 3, 7, 14 and 21 days after TBI) and sham control. Scale bar = 5 μm (**B1**–**B6**), Scale bar = 50 μm (**b1**–**b6**). **C** Images of BDNF^+^/GFAP ^+^/DAPI^+^ cells on day 7 post-CCI. Scale bar = 5 μm (**C1**), Scale bar = 50 μm (**c1**–**c2**). **D** The number of BDNF+/GFAP+/DAPI+ cells in cortex at different CCI time courses (1, 3, 7, 14 and 21 days after CCI) and sham control. **E1**, **e1** Images of DCX+/GFAP+ cells on day 7 post-CCI showing DCX+ and GFAP immunoreactivity as merged image. Scale bar = 25 μm (**E1**), Scale bar = 100 μm. (**e1**). **E2**, **e2** Images of BDNF+/DCX+ cells on day 7 post-CCI showing DCX+ and GFAP immunoreactivity as merged image. Scale bar = 25 μm (**E2**), Scale bar = 100 μm (**e2**). **E3**, **e3** Images of BDNF+/DCX+/GFAP+cells on day 7 post-CCI showing BDNF+, DCX+ and GFAP immunoreactivity as merged image. Scale bar = 25 μm (**E3**), Scale bar = 200 μm (**e3**, **e3-1**, **e3-2**, **e3-3**). The results revealed that the DCX-positive cells, BDNF-positive cells and GFAP-positive cells were accompanied at different time points post-CCI and showed a peak trend on day 7 post-CCI. Data are expressed as the mean ± SEM, *n* = 6 for each time-point. *, *P* < 0.05; one way ANOVA with Tukey’s multiple comparisons test. CCI: controlled cortical impact; TBI: traumatic brain injury; SVZ: subventricular zone; DAPI: 4,6-Diamidino-2-phenylindole; CC: corpus callosum

GFAP-positive cells in cortex increased on day 1 and 3 and showed a peak trend on day 7 (Fig. 3A). The GFAP-positive cells were still remarkably found on day 14 and on day 21, and there was a tendency of glial scar formation in the lesion area (Fig. 3A). BDNF-positive cells and GFAP-positive cells were co-located at different post-CCI time points (Fig. 3B, C) and the number of co-located cells peaked on day 7 post-CCI (*P* < 0. 05) (Fig. 3B–D). In addition, DCX positive cells, BDNF positive cells and GFAP positive cells were observed to be adjacent in the migrating path of neuroblasts after CCI (Fig. 3E).

### CCL2 promotes neuroblasts migration and reactive astrocytes-derived BDNF expression

**Fig. 4:**
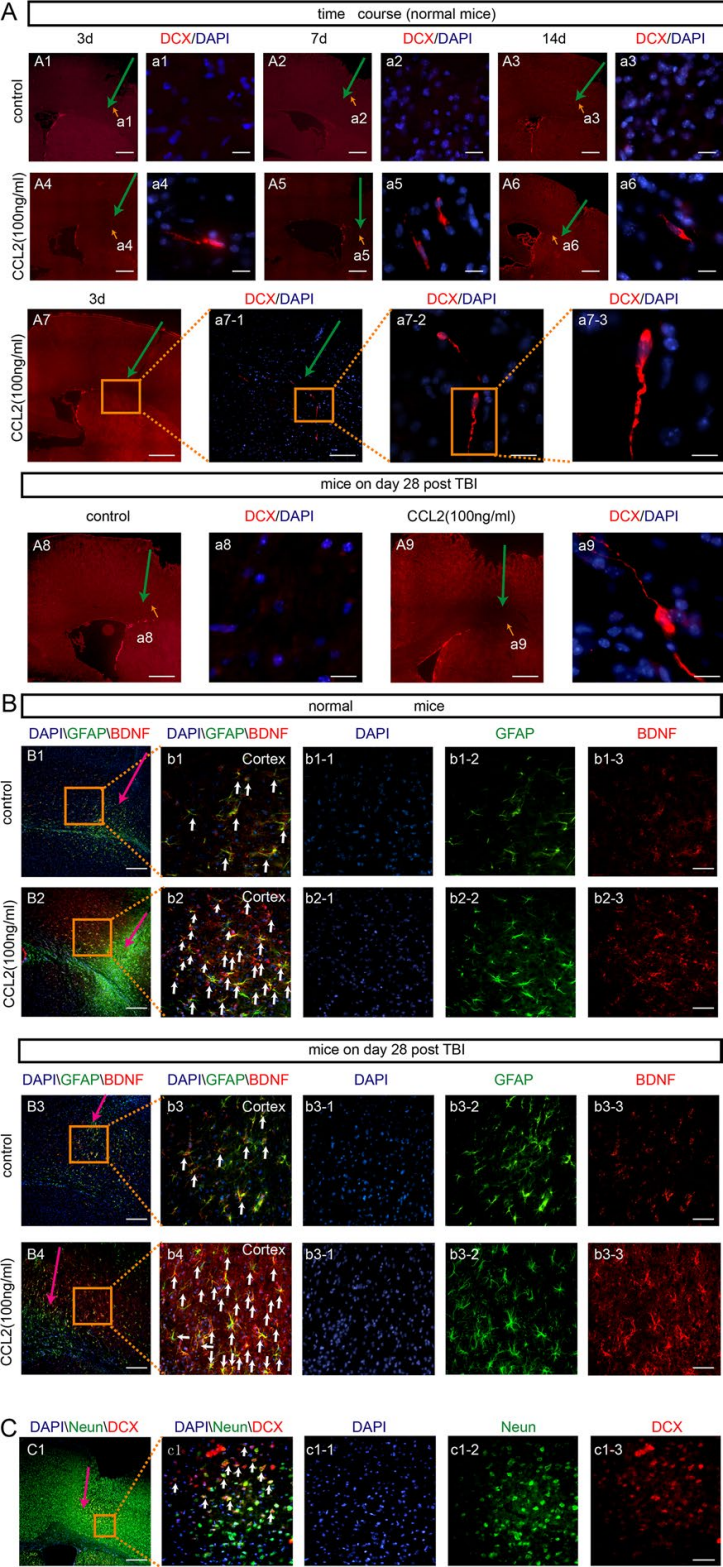
Exogenous CCL2 promoted neuroblasts migration and reactive astrocytes-derived BDNF expression in both normal and TBI mice (day 28). These provoked neuroblasts could differentiate into functional neurons. Coronal sections of forebrain were immunostained with anti-DCX antibody (red, neuroblasts), anti-BDNF antibody (red, BDNF-positive cells), anti-GFAP antibody (green, reactive astrocytes), anti-NeuN antibody (green, neurons) and DAPI (blue, nucleus). Yellow and pink arrows: injection pathway. **A1**–**A6**, **a1**–**a6** Photomicrograph showing the distribution of DCX^+^ cells at different injection time courses (3, 7 and 14 days after injection) in normal mice with vehicle or CCL2 (100 ng/mL). Scale bar = 5 μm (**A1**–**A6**), Scale bar = 200 μm (**a1**–**a6**). **A7**, **a7** Photomicrograph showing the distribution of DCX^+^ cells at 3 days after injection in normal mice with CCL2 (100 ng/mL). Scale bar = 5 μm (**A7**), 25 μm (**a7-1**), 50 μm (**a7-2**), and 200 μm (**a7-3**). **A9–A10**, **a9–a10** Photomicrograph showing the distribution of DCX^+^ cells on day 7 after injection in CCI mice with vehicle or CCL2 (100 ng/mL). Scale bar = 5 μm (**A9–A10**), 200 μm (**a9–a10**). **B1**, **b1** Images of BDNF+/GFAP+ cells in cortex of normal mice on day 7 post-injection with vehicle showing GFAP and BDNF immunoreactivity separately or as merged image. Scale bar = 12.5 μm (**B1**), 50 μm (**b1**). **B2**, **b2** Images of BDNF+/GFAP+ cells in cortex of normal mice on day 7 post-injection with CCL2 (100 ng/mL) showing GFAP and BDNF immunoreactivity separately or as merged image. Scale bar = 12.5 μm (**B2**), 50 μm (**b2**). **B3, b3** Images of BDNF+/GFAP+ cells in cortex of CCI 28d mice on day 7 post-injection with vehicle showing GFAP and BDNF immunoreactivity separately or as merged image. Scale bar = 12.5 μm (**B3**), 50 μm (**b3**). **B4, b4** Images of BDNF+/GFAP+ cells in cortex of CCI 28d mice on day 7 post-injection with CCL2 (100 ng/mL) showing GFAP and BDNF immunoreactivity separately or as merged image. Scale bar = 12.5 μm (**B4**), 50 μm (**b4**). **C** Images of DCX+/NeuN+/DAPI cells in cortex of normal mice on day 14 post-injection with CCL2 (100 ng/mL) showing NeuN and DCX immunoreactivity separately or as merged image. Scale bar = 5 μm (**C1**), 50 μm (**c1**). Pink arrow: injection track. CCI: controlled cortical impact; TBI: traumatic brain injury; CC chemokine ligand 2 = CCL2; DAPI: 4,6-Diamidino-2-phenylindole; CC: corpus callosum

Among the CCL2, TNF-*α*, IL-6 and IL-1*β*, CCL2 showed maximal effects to provoke neuroblast migration (unpublished data). The neuroblasts were discovered in SVZ, corpus callosum, striatum and cortex adjacent to the injection canal in the CCL2 groups (Fig. 4A4–A6, a4–a6). CCL2 could provoke neuroblast migration at three different concentrations (50 ng/mL, 100 ng/mL and 500 ng/mL), with 100 ng/mL being the optimal concentration (Fig. 4A7, a7-1, a7-2, a7-3). We injected CCL2 at concentration of 100 ng/mL into the cortex near lesion in mice on 28 days after CCI. Likewise, more DCX-positive cells were observed along the injection track in CCL2 group (Fig. 4A8–A9, a8–a9). Furthermore, both astrocytes number and BDNF expression were elevated by CCL2 treatment (Fig. 4B). Notably, DCX-positive cells were partially overlapped with NeuN within the injection track (Fig. 4C). In addition, the number of CCL2-positive cells and CCL2 level in CCI groups significantly increased compared with sham group. (*P* < 0.05), and CCL2 expression in cortex increased on day 1 and 3 post-CCI, peaking on day 7, and remained high on day 14 and on day 21 (Additional file 1: Fig. S2A–D).

### CCR2 antagonist restrains neuroblasts migration in TBI mice

**Fig. 5:**
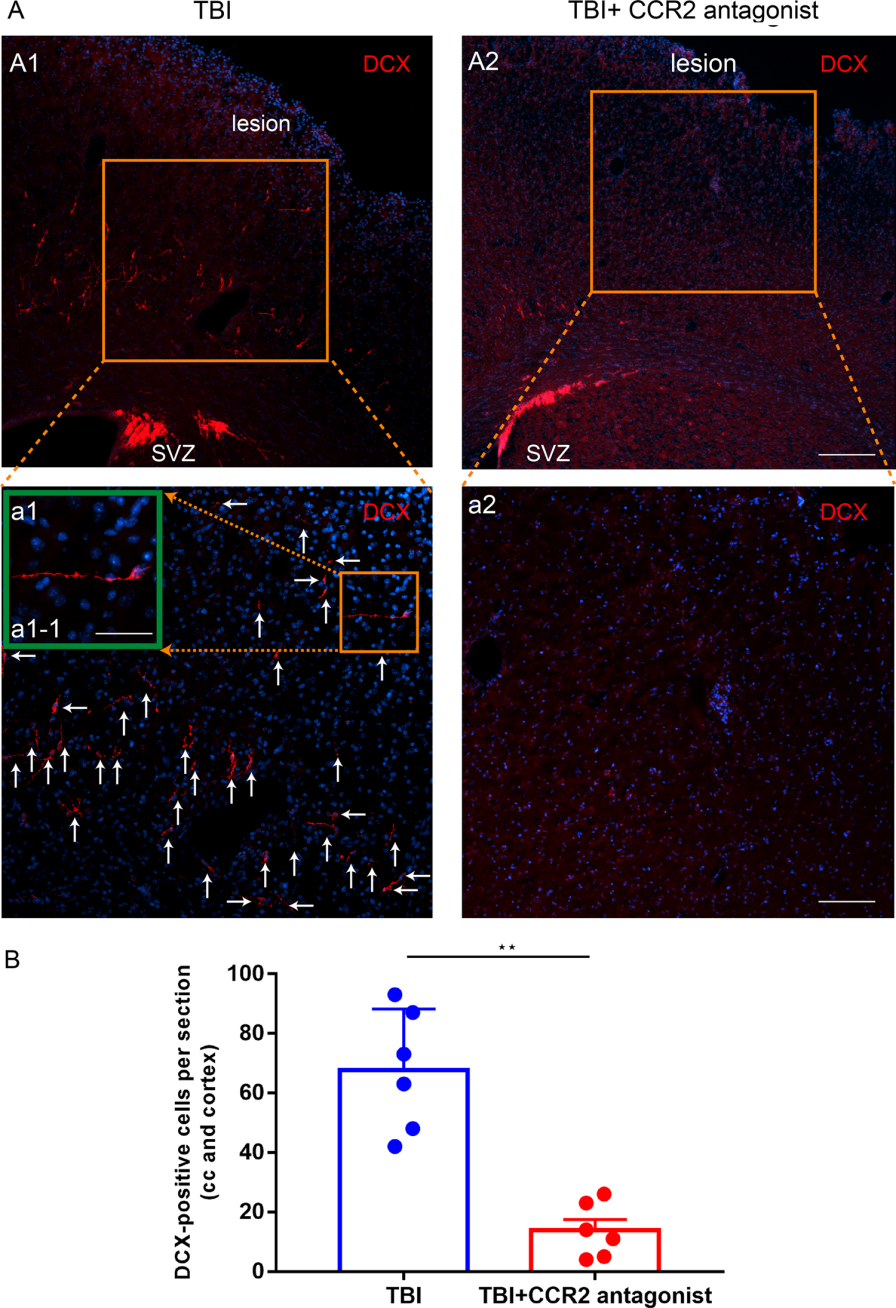
CCR2 antagonist restrained neuroblasts migration in TBI mice (day 7). Coronal sections of forebrain were immunostained with anti-DCX antibody (red, neuroblasts) and DAPI (blue, nucleus). White arrows: DCX^+^ cells. **A1, a1, a1-1** Photomicrograph showing the distribution of DCX^+^ cells in the TBI group. The DCX positive cells were found in SVZ, CC and injury cortex in the TBI-CCR2 antagonist group. Scale bar = 12.5 μm (**A1**), 25 μm (**a1**), 50 μm (**a1-1**). **A2, a2** Photomicrograph showing the distribution of DCX^+^ cells in the TBI-CCR2 antagonist group. The DCX positive cells were found only in SVZ and CC in the TBI group. Scale bar = 12.5 μm (**A2**), 25 μm (**a2**). **B** The number of migrating cells (DCX^+^ cells) in the CC and cortex was significantly smaller in the TBI-CCR2 antagonist group than in the TBI group (*P* < 0.05). Data are expressed as the mean ± SEM, *n* = 6 for each time-point. *, *P* < 0.05; Student’s *t*-test. CCI: controlled cortical impact; TBI: traumatic brain injury; CC chemokine ligand 2 = CCL2; DAPI: 4,6-Diamidino-2-phenylindole; CC: corpus callosum

The DCX positive cells were found in SVZ, CC and injured cortex in the TBI-CCR2 antagonist group, while they were found only in SVZ and CC in the TBI group (Fig. 5A). Moreover, the number of DCX-positive cells in the CC and cortex was significantly smaller in the TBI-CCR2 antagonist group than in the TBI group (*P* < 0.05, Fig. 5B). All the above results indicated that exogenous CCR2 antagonist can restrain the neuroblasts migration after TBI.

## Discussion

At present, there are no effective therapeutic strategies for severe neurological dysfunction in TBI patients. Numerous works have proven that stem cell therapy and neurogenesis can ameliorate brain injury in experimental models of TBI and other diseases [4–13]. However, the pattern and mechanism of endogenous neuroblasts migration after TBI is still unclear. We established a TBI model in mice and performed serial experiments to explore underlying mechanisms of injury-induced migration of neuroblasts.

Our results showed that neuroblasts migration initiated as early as day 1 after CCI, peaking on day 7, and persisted till day 21 post-CCI. According to the motile path, these immature precursors crossed CC and finally arrived at injured cortex on day 7 after TBI. On the basis of these findings, we investigated the following essential questions.

*Who regulates the orientation of neuroblasts from SVZ to cortex after TBI?* As these neural progenitors migrate in long distance throughout brain tissue, they are supported by various guidance cues as chemoattractants. For example, vascular endothelial cells secrete chemoattractive/trophic factors for neuroblasts, such as SDF-1, Ang1, and BDNF [13, 14, 43], which may help recruit neuroblasts to the vicinity of the vessels. Likewise, the astrocytes attract neuroblasts by secreting or trapping soluble factors including GABA and BDNF [9, 13, 14]. Moreover, BDNF was reported to control the direction of neural precursor cells movement or axon growth dependent on concentration difference [16, 17]. In this study, BDNF was highly expressed within migrating path, and the temporal characteristics of BDNF expression was similar to that of neuroblasts migration. More intriguingly, the level of BDNF expression was much higher in peri-lesion than in peri-SVZ after CCI. Thus, we speculate that the BDNF gradient is responsible for the navigation of neuroblasts after TBI.

*Who dominates the expression of BDNF within migrating path after TBI? BDNF* has been previously shown to be secreted from several cell types, i.e. neurons, astrocytes, microglia and endothelial cells after brain injury [13, 44]. In our study, reactive astrocyte was the major resource of BDNF in the early phase after CCI, distributed within the migrating path. In the late phase, BDNF expression was decreased despite high ratio of astrocytes on day 14 and 21 (glial scar), indicating that astrocytes in glial scar may be functionally inactive. In physical mice, chains of neuroblasts were surrounded by astrocytic tunnels called glial tubes, and neuroblasts migrated along glial tube [9]. In our study, reactive astrocytes were also distributed in migrating path following CCI. Moreover, DCX, BDNF and GFAP were adjacently expressed in this path, indicating that there was close relationship among neuroblasts, BDNF and reactive astrocytes. Our results indicated that reactive astrocytes could act as BDNF producer and migrating scaffold for neuroblasts migration.

*Who initiates and regulates above neuroblasts migration after TBI?* We attempted to seek potential regulators of neuroblasts migration with following criteria: polyergic control of astrocyte activation and BDNF secretion, and tested four cytokines (CCL2, TNF-1*α*, IL-6, and IL-1*β*). Among these cytokines, CCL2 exhibited maximal effects to promote neuroblasts migration, and possessed even similar spatiotemporal profile to BDNF expression, suggesting that CCL2 might be one of the endogenous regulators of neuroblasts migration. To verify the effects of exogenous chemoattractant in neuroblasts migration, the optimal concentration of CCL2 was injected into the different sites of hemisphere in normal mice. Consequently, it duplicated the neuroblasts migration from SVZ to injection sites and promoted local astrocytes activation and BDNF expression. Next, to test the effect of CCL2 in endogenous neuroblasts migration in the late phase of brain injury, CCL2 was injected into peri-lesion cortex in mice on day 28 after TBI. Interestingly, we observed a small number of neuroblasts could still cross glial scar and arrived at the lesion area. Notably, these neuroblasts also possessed the potential to differentiate into mature neurons. However, further studies are warranted regarding whether they finally work as functional neurons [11, 45, 46]. If the new mature neurons are functional and their formation can be stimulated, a novel therapeutic strategy might be developed for TBI in humans. In addition, CCR2 antagonist can restrain the neuroblasts migration after TBI. All the results indicated that exogenous CCL2 is promising to restart neuroblasts migration even in both normal and CCI mice (day 28; the late phase of brain injury). CCL2 is known to be up-regulated under various pathological conditions, including TBI, impairing the integrity of the blood–brain barrier (BBB) [47, 48]. CCL2 also initiates an inflammatory response after brain injury through recruitment of microglia and macrophage to the area of injury [49–51]. Whether and how these processes interfere with the endogenous mechanism of BDNF function and neuroblasts migration need further investigation. The capacity of neuroblasts migration is impaired under trauma condition, probably due to the detrimental microenvironment and glial scar obstruction [52–54], and optimal administrative protocol of CCL2 would be explored in the future work. Undoubtedly, we acknowledge that many other factors that were not considered here may influence neuroblast migration. In term of current mechanism, bioinformatic prediction and proteomic screening is warranted to seek more candidate targets in the future.

## Conclusions

The current study implicated a key step of neuroblasts migration following TBI: the neuroblasts migrate along the activated astrocytic network, directed by BDNF gradient between SVZ and injured cortex. Such process is under control of CCL2, which might act as a regulator of both spatial activation and BDNF secretion within astrocytes. Till now, implantation of exogenous stem cells in patients failed to pass the clinical trials. Thus, modulation of endogenous neurogenesis is regarded as a promising regimen for TBI therapy. Given CCL2 is a chemokine, early administration after TBI might trigger inflammatory response and aggravate brain injury. The strategy of delayed administration may provide a novel solution for late neurogenesis post-trauma. However, due to species differences between mice and humans, preclinical trials are needed before applied to clinic practice.

## Supplementary Information

**Additional file 1: Fig. S1.** Cellar source of BDNF expression after TBI. Coronal sections of forebrain were immunostained with anti-BDNF antibody (red, neuroblasts), anti-GFAP (green, astrocyte), anti-NeuN (green, neuron), anti-Iba1 (green, microglia), anti-CD31 (green, endothelial cells) and DAPI (blue, nucleus) to analyze cellar source of BDNF expression after CCI. (A): Images of BDNF+/GFAP+ cells in the peri-lesion cortex on day 7 post CCI showing BDNF (A2) and GFAP (A3) immunoreactivity separately or as merged image (A4). Scale bar = 50 μm. (B): Images of BDNF+/NeuN + cells in the peri-lesion cortex on day 7 post CCI showing BDNF (B2) and NeuN (B3) immunoreactivity separately or as merged image (B4) .Scale bar =50 μm. (C): Images of BDNF+/Iba1+ cells in the peri-lesion cortex on day 7 post CCI showing BDNF (C2) and Iba1 (C3) immunoreactivity separately or as merged image (C4).Scale bar =50 μm. (D): Images of BDNF+/CD31+ cells in the peri-lesion cortex on day 7 post CCI showing BDNF (D2) and CD31 (D3) immunoreactivity separately or as merged image (D4). Scale bar =50μm.(A-D) indicated that neurons, astrocytes, microglia and endothelial cells all contributed to BDNF expression after CCI. (E): The percentage of BDNF+/GFAP+ cells, BDNF+/NeuN+ cells, BDNF+/Iba1+ cells, and BDNF+/CD31+ cells in BDNF+ cells on day 7 post CCI. The results(a-e) indicated that astrocytes might be a major factor stimulating BDNF expression after CCI. Data are expressed as the mean ± SEM, n = 6 for each time-point. *, P < 0.05; one way ANOVA with Tukey’s multiple comparisons test. CCI: controlled cortical impact; TBI: traumatic brain injury; DAPI: 4,6-Diamidino-2-phenylindole. **Fig. S2.** Spatiotemporal characteristics of CCL2 expression after TBI. Coronal sections of forebrain were immunostained with anti-CCL2 antibody (red, CCL2-positive cells) and DAPI (blue, nucleus).The level of CCL2 in peri-lesion cortex was determined by CCL2/DAPI co-staining and ELISA analysis. (A): photomicrograph showing the distribution of CCL2+ cells at different CCI time courses (1, 3, 7, 14 and 21 days after CCI) and sham control. CCL2-positive cells in the cortex increased on day 1 post CCI, and significantly increased on day 3, with a peak expression on day 7 post CCI. The CCL2-positive cells showed a downward trend on day 14 and on day 21 post CCI. Scale bar = 5μm (A1-A6), Scale bar = 25μm(a1-a6). (B): Images of CCL2+/DAPI+ cells on day 3 and day 7 post CCI. (C): The number of migrating cells (CCL2+/DAPI+ cells) in peri-lesion cortex at different CCI time courses (1, 3, 7, 14 and 21 days after CCI) and sham control. The number of CCL2-positive cells increased markedly compared with the baseline (sham control) in peri-lesion area on 7 days post CCI. (D): ELISA analysis was applied to measure the concentration of CCL2 in peri-lesion cortex at different CCI time courses (1, 3, 7, 14 and 21 days after CCI) and sham control. Data are expressed as the mean ± SEM, n = 6 for each time-point. *, P < 0.05; one way ANOVA with Tukey’s multiple comparisons test. CCI: controlled cortical impact; TBI: traumatic brain injury; CC chemokine ligand 2=CCL2; DAPI: 4,6-Diamidino-2-phenylindole.

## Data Availability

All data generated during this study are included in this article and its Additional files.

## Abbreviations

Ang-1: Angiopoietin-1
BDNF: Brain derived neurotrophic factor
CC: Corpus callosum
CCI: Controlled cortical impact
DCX: Doublecortin
GFAP: Glial fibrillary acidic protein
CCL2: CC chemokine ligand 2
DAPI: 4,6-Diamidino-2-phenylindole
CC: Corpus callosum
MCP-1: Monocyte chemoattractant protein-1
CCR2: CC chemokine ligand receptor 2
MS: Rostral migratory stream
SDF-1*α*: Stromal cell derived factor-1 alpha
SGZ: Subgranular zone
SVZ: Subventricular zone
TBI: Traumatic brain injury
VEGF: Vascular endothelial growth factor
