# Supplementary material for: Neuroblasts migration under control of reactive astrocyte-derived BDNF: a promising therapy in late neurogenesis after traumatic brain injury
Source: Stem Cell Res Ther. 2023 Jan 5;14:2. doi: 10.1186/s13287-022-03232-0 (PMC9814466; doi:10.1186/s13287-022-03232-0)
Supplement: Supplementary file 1 — Additional file 1: Fig. S1. Cellar source of BDNF expression after TBI. Coronal sections of forebrain were immunostained with anti-BDNF antibody (red, neuroblasts), anti-GFAP (green, astrocyte), anti-NeuN (green, neuron), anti-Iba1 (green, microglia), anti-CD31 (green, endothelial cells) and DAPI (blue, nucleus) to analyze cellar source of BDNF expression after CCI. (A): Images of BDNF+/GFAP+ cells in the peri-lesion cortex on day 7 post CCI showing BDNF (A2) and GFAP (A3) immunoreactivity separately or as merged image (A4). Scale bar = 50 μm. (B): Images of BDNF+/NeuN + cells in the peri-lesion cortex on day 7 post CCI showing BDNF (B2) and NeuN (B3) immunoreactivity separately or as merged image (B4) .Scale bar =50 μm. (C): Images of BDNF+/Iba1+ cells in the peri-lesion cortex on day 7 post CCI showing BDNF (C2) and Iba1 (C3) immunoreactivity separately or as merged image (C4).Scale bar =50 μm. (D): Images of BDNF+/CD31+ cells in the peri-lesion cortex on day 7 post CCI showing BDNF (D2) and CD31 (D3) immunoreactivity separately or as merged image (D4). Scale bar =50μm.(A-D) indicated that neurons, astrocytes, microglia and endothelial cells all contributed to BDNF expression after CCI. (E): The percentage of BDNF+/GFAP+ cells, BDNF+/NeuN+ cells, BDNF+/Iba1+ cells, and BDNF+/CD31+ cells in BDNF+ cells on day 7 post CCI. The results(a-e) indicated that astrocytes might be a major factor stimulating BDNF expression after CCI. Data are expressed as the mean ± SEM, n = 6 for each time-point. *, P < 0.05; one way ANOVA with Tukey’s multiple comparisons test. CCI: controlled cortical impact; TBI: traumatic brain injury; DAPI: 4,6-Diamidino-2-phenylindole. Fig. S2. Spatiotemporal characteristics of CCL2 expression after TBI. Coronal sections of forebrain were immunostained with anti-CCL2 antibody (red, CCL2-positive cells) and DAPI (blue, nucleus).The level of CCL2 in peri-lesion cortex was determined by CCL2/DAPI co-staining and ELISA analysis. (A): photomicrograph show [file 13287_2022_3232_MOESM1_ESM.docx]

**Supplementary**

**Figure S1.** **Cellar source of BDNF expression after TBI.**

**
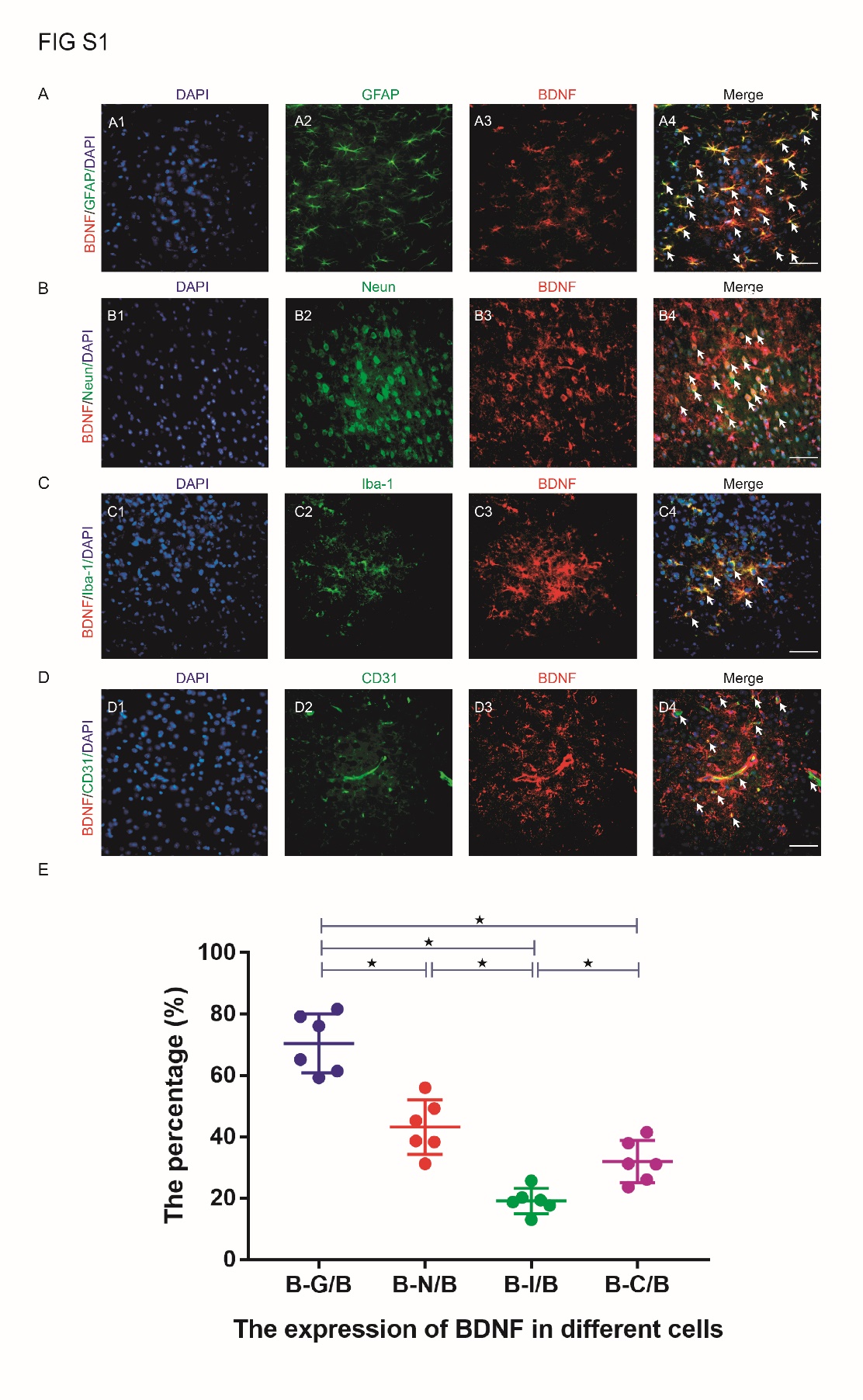
**

Coronal sections of forebrain were immunostained with anti-BDNF antibody (red, neuroblasts), anti-GFAP (green, astrocyte), anti-NeuN (green, neuron), anti-Iba1 (green, microglia), anti-CD31 (green, endothelial cells) and DAPI (blue, nucleus) to analyze cellar source of BDNF expression after CCI. (A): Images of BDNF+ /GFAP+ cells in the peri-lesion cortex on day 7 post CCI showing BDNF (A2) and GFAP (A3) immunoreactivity separately or as merged image (A4). Scale bar = 50 μm. (B): Images of BDNF+ / NeuN + cells in the peri-lesion cortex on day 7 post CCI showing BDNF (B2) and NeuN (B3) immunoreactivity separately or as merged image (B4) .Scale bar =50 μm. (C): Images of BDNF+ / Iba1+ cells in the peri-lesion cortex on day 7 post CCI showing BDNF (C2) and Iba1 (C3) immunoreactivity separately or as merged image (C4).Scale bar =50 μm. (D): Images of BDNF+ / CD31+ cells in the peri-lesion cortex on day 7 post CCI showing BDNF (D2) and CD31 (D3) immunoreactivity separately or as merged image (D4). Scale bar =50μm.(A-D) indicated that neurons, astrocytes, microglia and endothelial cells all contributed to BDNF expression after CCI. (E): The percentage of BDNF+ /GFAP+ cells, BDNF+ /NeuN+ cells, BDNF+ /Iba1+ cells, and BDNF+ /CD31+ cells in BDNF+ cells on day 7 post CCI. The results(a-e) indicated that astrocytes might be a major factor stimulating BDNF expression after CCI. Data are expressed as the mean ± SEM, n = 6 for each time-point. *, P < 0.05; one way ANOVA with Tukey’s multiple comparisons test. CCI: controlled cortical impact; TBI: traumatic brain injury; DAPI: 4,6-Diamidino-2-phenylindole.

**Figure S2. Spatiotemporal characteristics of** CCL2 **expression after TBI.**

**
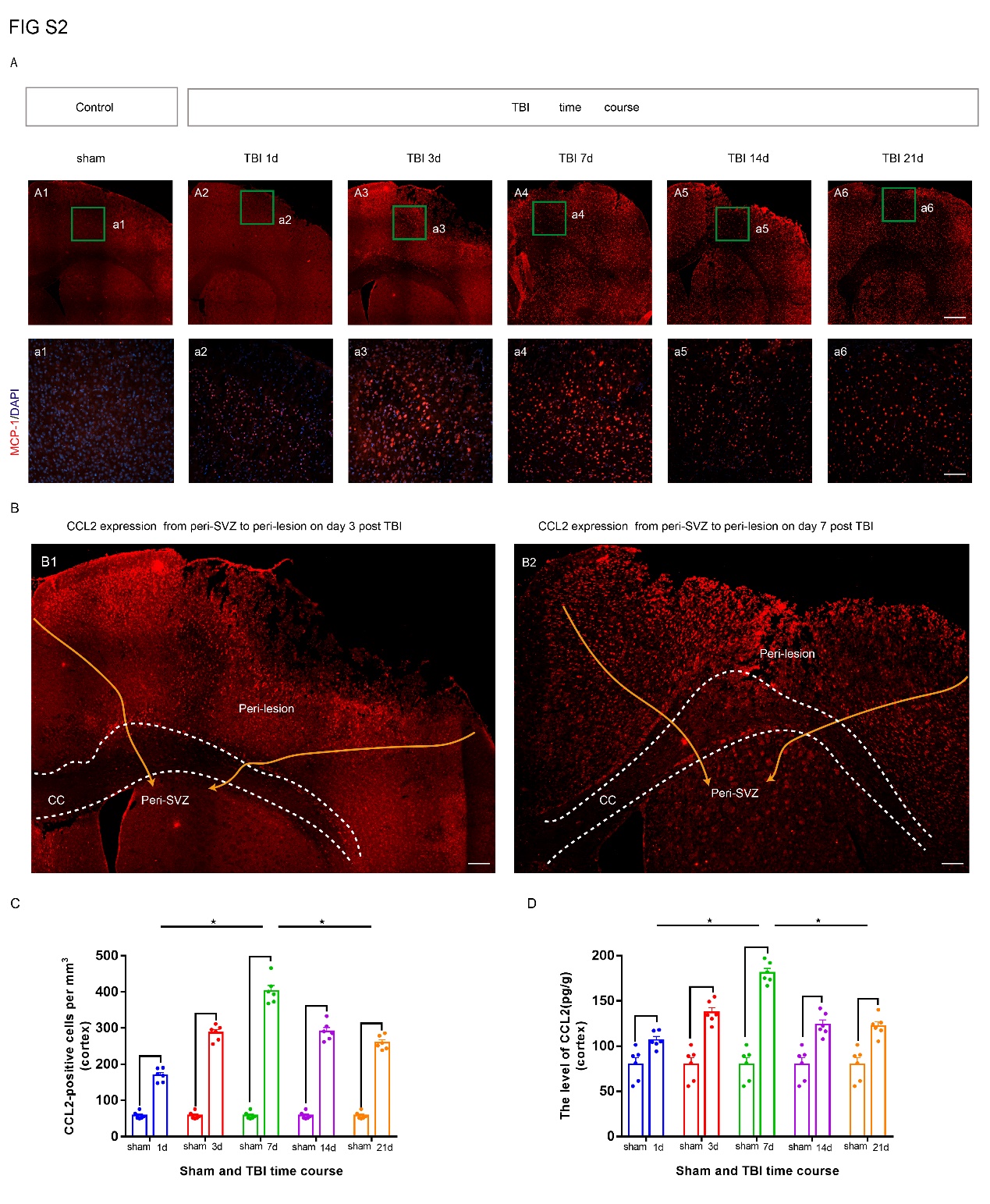
**

Coronal sections of forebrain were immunostained with anti-CCL2 antibody (red, CCL2-positive cells) and DAPI (blue, nucleus).The level of CCL2 in peri-lesion cortex was determined by CCL2/ DAPI co-staining and ELISA analysis. (A): photomicrograph showing the distribution of CCL2+ cells at different CCI time courses (1, 3, 7, 14 and 21days after CCI) and sham control. CCL2-positive cells in the cortex increased on day 1 post CCI, and significantly increased on day 3, with a peak expression on day 7 post CCI. The CCL2-positive cells showed a downward trend on day 14 and on day 21 post CCI. Scale bar = 5μm (A1-A6), Scale bar = 25μm(a1-a6). (B): Images of CCL2+ /DAPI+ cells on day 3 and day 7 post CCI. (C): The number of migrating cells (CCL2+ / DAPI+ cells) in peri-lesion cortex at different CCI time courses (1, 3, 7, 14 and 21days after CCI) and sham control. The number of CCL2-positive cells increased markedly compared with the baseline (sham control) in peri-lesion area on 7 days post CCI. (D): ELISA analysis was applied to measure the concentration of CCL2 in peri-lesion cortex at different CCI time courses (1, 3, 7, 14 and 21days after CCI) and sham control. Data are expressed as the mean ± SEM, n = 6 for each time-point. *, P < 0.05; one way ANOVA with Tukey’s multiple comparisons test. CCI: controlled cortical impact; TBI: traumatic brain injury; CC chemokine ligand 2=CCL2; DAPI: 4,6-Diamidino-2-phenylindole.
